# Associations between smoking and osteoporosis and all-cause mortality in participants from the United States: a cohort study

**DOI:** 10.3389/fendo.2025.1533633

**Published:** 2025-03-24

**Authors:** Xiaoqin Qu, Jingcheng Jiang, Qingshan Deng, Han Wang, Chao Zhang, Xiaoping Xu, Yong Yi, Lihua Qiu

**Affiliations:** ^1^ Department of Radiology, The Second People’s Hospital of Yibin, Clinical Research and Translational Center, Neuroimaging Big Data Research Center, The Second People’s Hospital of Yibin, Yibin, China; ^2^ Department of Neurosurgery, The Second People’s Hospital of Yibin, Yibin, China

**Keywords:** smoke, osteoporosis, interaction, all-cause mortality, NHANES

## Abstract

**Background:**

Smoking is a global public health concern, with approximately 1,245 billion smokers worldwide. It is associated with a range of health complications, including cardiovascular and respiratory diseases. Osteoporosis, characterized by reduced bone density and deterioration of bone tissue, has a global prevalence of 18.3%, with higher rates in women over the age of 50. Smoking has been recently associated with osteoporosis, potentially due to shared metabolic disorders or personal habits. This study aimed to investigate the association between smoking and osteoporosis in relation to all-cause mortality in a cohort from the United States.

**Methods:**

Data were sourced from the National Health and Nutrition Examination Survey (NHANES) database, which focuses on individuals aged 20 years and older from 2005–2010, 2013–2014, and 2017–2018, where femoral neck bone density testing was conducted. The participants were categorized on the basis of their self-reported smoking status and bone mineral density (BMD) measurements, following the World Health Organization criteria for osteoporosis. The covariates included age, sex, race, alcohol consumption, BMI, blood glucose levels, and other health indicators. Statistical analysis included ANOVA and chi-square tests for baseline characteristics, Kaplan–Meier survival analysis, and multivariate Cox regression analysis to assess hazard ratios (HRs) and 95% confidence intervals (CIs) for all-cause mortality. We divided the patients into four different groups via a cross-classification method on the basis of smoking status and whether they had osteoporosis.

**Results:**

This study included 19,400 participants, with significant differences in baseline characteristics across 4 groups (S-/OP+: nonsmokers with osteoporosis; S+/OP-: smokers without osteoporosis; S-/OP-: nonsmokers without osteoporosis; S+/OP+: smokers with osteoporosis). The overall average age was 53.1 years, and women accounted for 49.6% of the total population. The mortality rate due to all factors in the total population was 13.1%, with the highest S+/OP+ mortality rate. Participants with both a smoking history and osteoporosis had a 146% increase in all-cause mortality (HR: 2.46, 95% CI: 2.12–2.87) even after adjusting for confounding factors. The relative excess risk due to interaction (RERI) suggested a lack of statistical significance, whereas the attributable proportion (AP) indicated a synergistic effect between smoking and osteoporosis.

**Conclusions:**

This cohort study highlights the importance of managing and preventing smoking and osteoporosis to reduce the risk of all-cause mortality. The findings provide preliminary evidence of a synergistic effect between smoking and osteoporosis on all-cause mortality risk, emphasizing the need for proactive strategies for smoking cessation and close monitoring of risk factors in individuals with both conditions.

## 1 Introduction

Globally, smoking is recognized as a significant public health issue. According to the World Health Organization, the global estimate of smokers reached approximately 1,245 billion individuals in 2022 (1). Osteoporosis, characterized by decreased bone density and deterioration of bone tissue microstructure, has an estimated global prevalence of 18.3%. Among individuals aged 50 and above, the prevalence of osteoporosis is markedly higher in women (33%) than in men (20%) (2). The adverse health effects of smoking are multifaceted, affecting various organs and leading to complications such as cardiovascular and respiratory diseases (3). Similarly, osteoporosis poses considerable risks, manifesting in the weakening and fragility of the musculoskeletal system and increasing the risk of fractures. Both conditions contribute significantly to disease-related complications in the general population. Recent studies have revealed a correlation between smoking and osteoporosis, suggesting potential coexistence due to metabolic disorders or personal habits and possibly a causal relationship in their progression (4). Smoking directly impacts bone metabolism and strength, leading to the deterioration of bone microarchitecture and an increased risk of osteoporosis (5). The increased incidence of osteoporosis associated with smoking has become a significant concern. However, the extent to which their coexistence influences patient prognosis remains uncertain. This study aimed to investigate the potential interaction between smoking and osteoporosis in relation to all-cause mortality when both conditions are present.

## 2 Materials and methods

### 2.1 Research population

The data for this study were extracted from the National Health and Nutrition Examination Survey (NHANES) database, a comprehensive survey conducted by the National Center for Health Statistics (NCHS) under the Centers for Disease Control and Prevention (CDC) in the United States (6). The NHANES evaluates the health and nutritional status of the American population, employing a combined approach of interviews and physical examinations. This extensive, probability-based survey is conducted annually over a 2-year cycle (7). The retrospective analysis in this study utilized data from five specific NHANES cycles (2005–2010, 2013–2014, and 2017–2018), as these were the only cycles that included femoral neck bone density testing. The NCHS Research Ethics Review Committee ensures that informed consent is obtained from all participants. For detailed statistical information, please refer to the NHANES website (https://www.cdc.gov/nchs/hanes/) (6). This cohort study included a sample of 19,400 individuals aged 20 years and older.

### 2.2 Assessment of tobacco exposure

During family interviews, individuals aged 20 years and older were asked to self-report their smoking status. Participants who reported having smoked fewer than 100 cigarettes in their lifetime were classified as “never smokers.” Those who had smoked more than 100 cigarettes at some point in their lives but had since quit smoking were identified as “former smokers.” Conversely, individuals who continued to smoke were considered “current smokers” (4). Former smokers and current smokers are classified as smokers.

### 2.3 BMD measurements and definitions of osteopenia and osteoporosis

The diagnosis of osteoporosis in this study was based on the criteria set by the World Health Organization, which defines the condition as a bone mineral density at the femoral neck that is 2.5 or more standard deviations below the mean for young adults of the same sex. Specifically, a T score of ≤–2.5 indicates osteoporosis, a T score between –2.5 and –1 indicates osteopenia (bone loss), and a T score >–1 is considered normal (8, 9).

**Figure 1:**
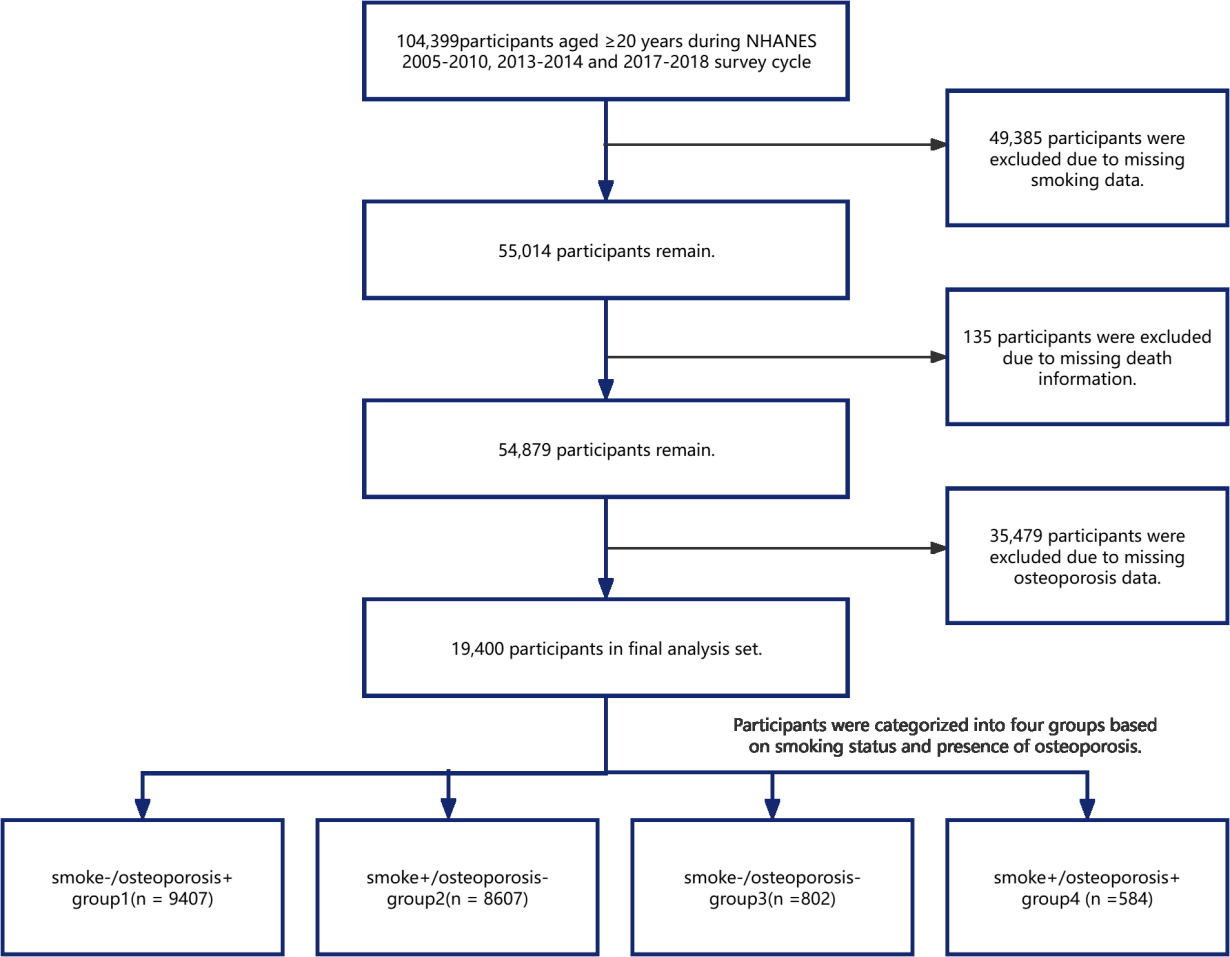
Flowchart of the study design.

Participants with reduced bone mass, classified as having osteopenia, and those with normal bone density were grouped as nonosteoporotic. The study excluded individuals without smoking information (n=49,385), those without an osteoporosis diagnosis (n=35,479), and 135 individuals with incomplete mortality data. The final sample comprised 19,400 participants. These participants were categorized into four distinct groups on the basis of their smoking status and the presence or absence of osteoporosis via a cross-classification approach. S-/OP+: Participants who never smoked and had osteoporosis (T score ≤ -2.5). S+/OP-: Participants who smoked (current or former smokers) and did not have osteoporosis (T score > -2.5). S-/OP-: Participants who never smoked and did not have osteoporosis (T score > -2.5). S+/OP+: Participants who smoked (current or former smokers) and had osteoporosis (T score ≤ -2.5) (**Figure 1**).

### 2.4 Covariates

In the National Health and Nutrition Examination Survey (NHANES), data collection is conducted through standardized questionnaires administered during family interviews, complemented by medical assessments for each participant. The covariates considered in this study included age, sex, race, alcohol consumption, body mass index (BMI), blood glucose levels, albumin, calcium, phosphorus, triglycerides, cholesterol, high-density lipoprotein (HDL), self-reported diabetes, and self-reported hypertension. Historical smoking referred to individuals who had smoked 100 or more cigarettes (“smokers”) or those who had smoked fewer than 100 cigarettes (“nonsmokers”). Drinking status was used to categorize participants as “nondrinkers” or “drinkers.” On the basis of self-reported diabetes status, the participants were classified as those with or without diabetes. Similarly, on the basis of self-reported hypertension, individuals were grouped into those with and without hypertension. The BMI of the participants was as follows: normal weight, 18.5–24.9 kg/m²; overweight, between 25.0 and 29.9 kg/m²; and obese, 30.0 kg/m² or higher. A detailed description of each variable used in the statistical analysis can be found online.^1^

### 2.5 Statistical analysis

Continuous variables are presented as the means ± standard errors (SDs), whereas categorical variables are expressed as counts with corresponding percentages. Baseline characteristics were analyzed via analysis of variance (ANOVA) for continuous variables and the chi-square test for categorical variables.

The survival patterns of different subgroups were evaluated through Kaplan–Meier analysis, which generated comparative survival curves. Multivariate Cox proportional hazards regression modeling was subsequently performed to quantify mortality risk. Specifically, we calculated hazard ratios (HRs) with corresponding 95% confidence intervals (CIs) for three experimental groups—S+/OP-, S-/OP-, and S+/OP+—using S-/OP+ participants as the reference group for all-cause mortality risk assessment. Model 1 was unadjusted for any confounding variables. Model 2 was adjusted for age, sex, and race. Model 3 included additional adjustments for age (<65 vs. ≥65 years), sex (male/female), race (non-Hispanic White/Black/Other), BMI (normal/overweight/obese), alcohol consumption (yes/no), diabetes mellitus (yes/no), and hypertension (yes/no). Furthermore, stratified analyses were performed on the basis of subgroups defined by age, sex, race, BMI, diabetes mellitus, and hypertension to assess potential differences between these subgroups. To explore the synergistic effect between smoking and osteoporosis, the relative excess risk due to interaction (RERI), the attributable proportion (AP) of interactions, and the corresponding 95% CIs were calculated.

All the data were analyzed via R^2^ (version 4.2.2, The R Foundation) and the Free Statistical analysis platform (version 2.0, Beijing, China). A p value of less than 0.05 was considered statistically significant.

## 3 Results

### 3.1 Baseline characteristics

The baseline characteristics varied among the exposed groups (**Table 1**). The average age across the population was 53.1 years, with S-/OP- individuals exhibiting the highest average age (68.2 years) and S-/OP+ individuals the lowest (50.4 years), p<0.001. Women constituted 49.6% of the total population, with S-/OP- having the highest proportion (84.8%), followed by S+/OP+ (65.8%), p<0.001. The prevalence of non-Hispanic white individuals was notably high (p<0.001).

**Table 1:** Baseline study population characteristics.

Variables	Total (n = 19400)	S-/OP+ (n = 9407)	S+/OP- (n = 8607)	S-/OP- (n = 802)	S+/OP+ (n = 584)	p
Age, Mean ± SD	53.1 ± 16.9	50.4 ± 16.9	53.6 ± 16.4	68.2 ± 12.9	67.5 ± 11.2	< 0.001
Sex, n (%)						< 0.001
male	9773 (50.4)	4104 (43.6)	5347 (62.1)	122 (15.2)	200 (34.2)	
female	9627 (49.6)	5303 (56.4)	3260 (37.9)	680 (84.8)	384 (65.8)	
Race, n (%)						< 0.001
Non-Hispanic White	9061 (46.7)	3777 (40.2)	4524 (52.6)	385 (48)	375 (64.2)	
Non-Hispanic Black	3875 (20.0)	1998 (21.2)	1763 (20.5)	61 (7.6)	53 (9.1)	
Other Race	6464 (33.3)	3632 (38.6)	2320 (27)	356 (44.4)	156 (26.7)	
BMI, n (%)						< 0.001
18.5~24.99 kg/m2	5565 (28.7)	2445 (26)	2373 (27.6)	424 (52.9)	323 (55.3)	
25.00~29.9 kg/m2	6897 (35.6)	3352 (35.6)	3120 (36.2)	253 (31.5)	172 (29.5)	
≥30.00 kg/m2	6938 (35.8)	3610 (38.4)	3114 (36.2)	125 (15.6)	89 (15.2)	
Drink, n (%)						< 0.001
no	7225 (37.2)	3814 (40.5)	2621 (30.5)	522 (65.1)	268 (45.9)	
yes	12175 (62.8)	5593 (59.5)	5986 (69.5)	280 (34.9)	316 (54.1)	
DM, n (%)						< 0.001
no	15542 (80.1)	7656 (81.4)	6806 (79.1)	622 (77.6)	458 (78.4)	
yes	3858 (19.9)	1751 (18.6)	1801 (20.9)	180 (22.4)	126 (21.6)	
Hypertension, n (%)						< 0.001
no	10476 (54.0)	5447 (57.9)	4484 (52.1)	313 (39)	232 (39.7)	
yes	8924 (46.0)	3960 (42.1)	4123 (47.9)	489 (61)	352 (60.3)	
PFG mmol/L, Mean ± SD	5.8 ± 2.2	5.7 ± 2.2	5.8 ± 2.2	5.9 ± 2.1	5.8 ± 1.9	0.008
ALB g/L, Mean ± SD	42.1 ± 3.2	42.2 ± 3.1	42.1 ± 3.2	41.6 ± 2.9	41.2 ± 3.5	< 0.001
P mmol/L, Mean ± SD	1.2 ± 0.2	1.2 ± 0.2	1.2 ± 0.2	1.2 ± 0.2	1.2 ± 0.2	< 0.001
Ca2^+^ mmol/L, Mean ± SD	2.4 ± 0.1	2.4 ± 0.1	2.4 ± 0.1	2.4 ± 0.1	2.4 ± 0.1	0.16
TG mmol/L, Mean ± SD	1.8 ± 1.5	1.7 ± 1.5	1.9 ± 1.6	1.6 ± 1.0	1.7 ± 1.0	< 0.001
TC mmol/L, Mean ± SD	5.1 ± 1.1	5.1 ± 1.0	5.0 ± 1.1	5.2 ± 1.1	5.2 ± 1.2	< 0.001
HDL mmol/L, Mean ± SD	1.4 ± 0.4	1.4 ± 0.4	1.3 ± 0.4	1.5 ± 0.4	1.5 ± 0.5	< 0.001
all_cause_mort, n (%)	2538 (13.1)	764 (8.1)	1336 (15.5)	207 (25.8)	231 (39.6)	< 0.001

With respect to the BMI distribution, 28.7% of the participants were within 18.5–24.99 kg/m², with S+/OP+ accounting for the highest proportion (55.3%) and S-/OP+ accounting for the lowest proportion (26%), p<0.001. The proportion of participants who consumed alcohol was 62.8%, with S+/OP- having the highest proportion (69.5%) and S-/OP- having the lowest (34.9%), p<0.001. Among the participants, 19.9% had diabetes, with the lowest proportion of S-/OP+ patients (18.6%) and the highest proportion of S-/OP- patients (22.4%), p<0.001. Hypertension was present in 46.0% of the participants, with the lowest proportion found in the S-/OP+ group (42.1%) and the highest in the S-/OP- group (61%), p<0.001.

The serum calcium levels did not differ significantly among the groups (p > 0.05). The overall mortality rate attributed to all causes was 13.1%. S+/OP+ had the highest mortality rate at 39.6%, whereas S-/OP+ had the lowest mortality rate at 8.1%. S-/OP+ and S-/OP- had mortality rates of 15.5% and 25.8%, respectively.

### 3.2 Associations with all-cause mortality

**Figure 2:**
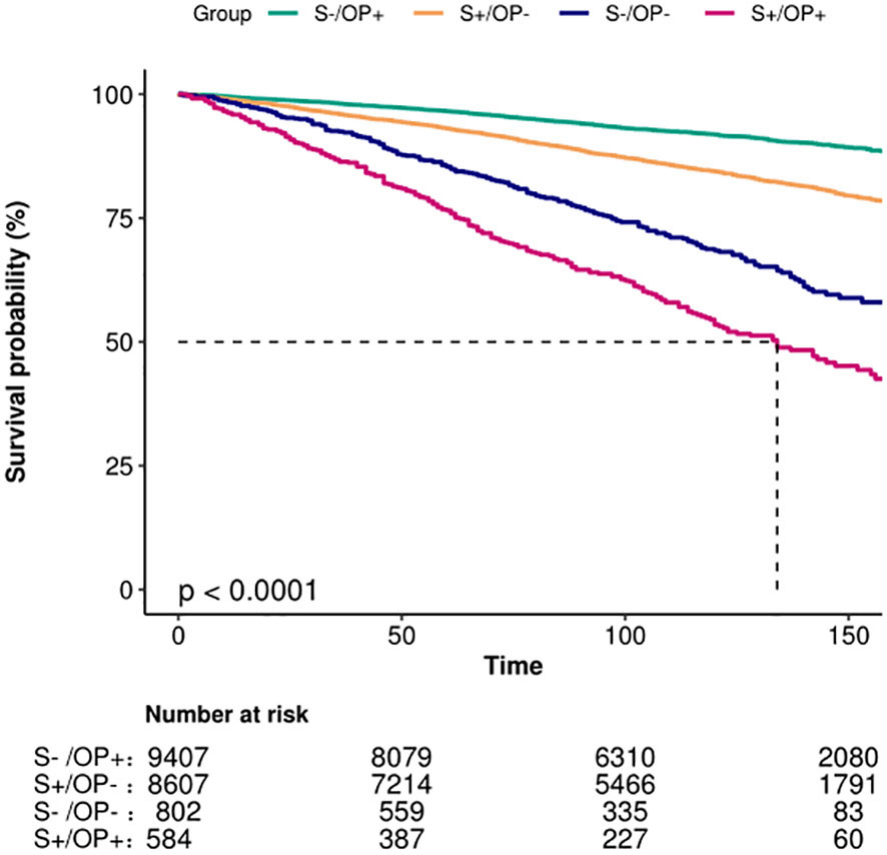
Kaplan–Meier survival estimates for all-cause mortality.

The Kaplan–Meier survival analysis indicated that patients who were S-/OP+ had the highest survival rate. Those with S+/OP - and S-/OP - followed in descending order. Notably, the S+/OP+ group presented the lowest survival rate (p<0.001, as shown in **Figure 2**). The higher mortality rate in the S-/OP- group (nonsmokers without osteoporosis) than in the other groups is likely due to the greater proportion of older individuals in the S-/OP group. Age is a well-known risk factor for mortality. In all the models, participants who smoked had osteoporosis, or both were at greater risk of all-cause mortality than those who neither smoked nor had osteoporosis (**Table 2**). Although this association was somewhat reduced after adjusting for various confounding factors, it remained statistically significant. According to the fully adjusted model, the all-cause mortality rate for individuals with both smoking and osteoporosis increased by 1.46% (HR: 2.46, 95% CI: 2.12–2.87). Across all the models, the associations showed a consistent pattern, with the most pronounced impact observed among patients who smoked and those with osteoporosis. In contrast, for patients diagnosed with osteoporosis alone, the effect was minimal.

**Table 2:** Association between smoking and osteoporosis and all-cause death.

Variable	Model1		Model2		Model3	
	HR(95%CI)	p_value	HR(95%CI)	p_value	HR(95%CI)	p_value
S-/OP+	1(Ref)		1(Ref)		1(Ref)	
S+/OP-	1.97 (1.8~2.16)	<0.001	1.54 (1.4~1.69)	<0.001	1.49 (1.36~1.63)	<0.001
S-/OP-	4.35 (3.73~5.07)	<0.001	1.46 (1.24~1.71)	<0.001	1.45 (1.24~1.71)	<0.001
S+/OP+	7.08 (6.11~8.21)	<0.001	2.66 (2.29~3.1)	<0.001	2.46 (2.12~2.87)	<0.001
Additive interaction						
RERI (95%CI) for smoke and osteoporosis:					-0.31(-0.6- -0.02)	0.98
AP (95%CI) for smoke and osteoporosis:					-0.31(-0.58- -0.04)	0.01

Model 1: Not adjusted.

Model 2: Adjusted for age, sex, race, BMI, alcohol consumption, DM, and hypertension.

Model 3: Adjusted by age, sex, race, BMI, drinking, DM, hypertension, PFG, ALB, P, Ca2+, TG, TC, and HDL.

### 3.3 Additive interaction

This study specifically aimed to investigate the interaction between smoking and osteoporosis. The relative excess risk due to interaction (RERI) was calculated as –0.31 (95% CI: –0.6–0.02, p = 0.98), which suggests that there is no statistically significant interaction effect. Conversely, the 95% CI for the attributable proportion (AP) extended from –0.31 to –0.04 (p = 0.01), indicating a significant synergistic effect between smoking and osteoporosis (**Table 2**).

### 3.4 Subgroup analysis

**Figure 3:**
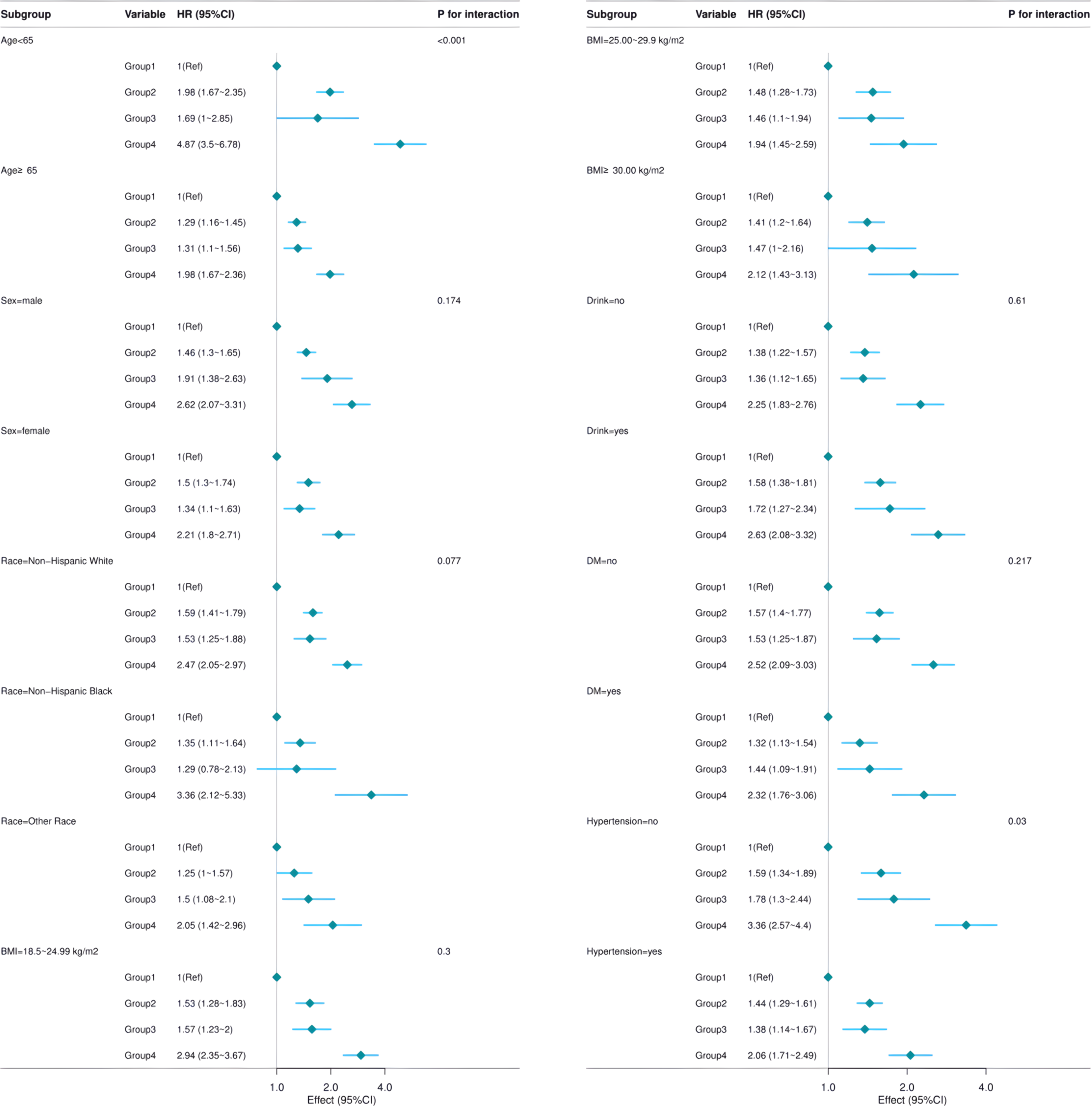
Subgroup analysis.

Covariates were selected on the basis of prior evidence, biological relevance, and NHANES data availability. The subgroups were stratified by age, sex, race, BMI, alcohol use, diabetes, and hypertension. In this stratified analysis, patients who both smoked and had osteoporosis were found to have the highest relative risk of mortality compared with those who neither smoked nor had osteoporosis. However, when stratification was based on the presence of hypertension, no significant difference in mortality rates was observed (**Figure 3**).

## 4 Discussion

In this cohort study, we identified significant disparities in baseline characteristics across various groups, which may influence the risk of all-cause mortality. Notably, an increase in age was associated with a heightened risk of all-cause mortality, aligning with established epidemiological trends (10–12). Furthermore, our findings corroborate the elevated mortality risk linked to the concurrent presence of smoking and osteoporosis. The combined impact of smoking and osteoporosis on all-cause mortality was found to exceed the anticipated effects of each factor independently. This association persisted even after controlling various confounding variables. Specifically, after adjusting for age, sex, race, BMI, and diabetes mellitus status, the results remained consistent.

While osteoporosis has traditionally not been classified as a complication of smoking, emerging evidence indicates an increasing prevalence of osteoporosis among smokers (4, 13). The chronic nature of these conditions is linked to elevated incidence rates, increased mortality, and substantial social costs. Epidemiological research has demonstrated a correlation between smoking and an elevated risk of fractures. Fractures related to osteoporosis, particularly those affecting the spine and hip, frequently result in chronic pain, disability, and a diminished quality of life. Such fractures necessitate hospitalization, increase the mortality risk by 20%, and result in chronic disability in up to 50% of cases (14–16). The complications associated with smoking further aggravate fracture outcomes. Consequently, it is imperative to pay increased attention to the coexistence of these two conditions. Nevertheless, few studies have examined the combined impact of smoking and osteoporosis on all-cause mortality. Smoking contributes to an increase in all-cause mortality. With this finding, current smokers have a higher mortality rate than nonsmokers do. It is possible to reduce health risks by quitting smoking at a young age (17–19).

Our study revealed that, within fully adjusted models, participants with both a smoking history and osteoporosis presented a 146% increased risk of all-cause mortality (HR: 2.46, 95% CI: 2.12–2.87), exceeding the risk associated with either smoking or osteoporosis alone (**Table 2**). These findings are consistent with the results of the additive interaction analysis (AP = -0.31, p = 0.01), which strongly suggests a synergistic effect. This implies that shared biological pathways may contribute to these unfavorable outcomes. Diabetes mellitus and hypertension are posited to be potentially significant factors in this context (20–22).

Considering the interaction between smoking and osteoporosis, we hypothesized that the observed increase in mortality risk among patients with both conditions was not simply an additive consequence of the individual risks associated with each condition. The results from our additive interaction model suggest a synergistic effect between smoking and osteoporosis. Our findings indicate a potential interaction between these two conditions, which may contribute to an elevated overall mortality risk.

The deleterious effects of smoking on bone health are likely due to multiple biological mechanisms. Primarily, smoking directly affects bone metabolism by inhibiting osteoblast differentiation and concurrently enhancing osteoclast activity (23, 24). Additionally, smoking may further reduce bone density through its impact on hormonal regulation, particularly by altering estrogen levels (25). The inflammatory response and oxidative stress induced by smoking can adversely affect bone health. These biological mechanisms may help explain the increased risk of osteoporosis and all-cause mortality observed in smokers. Consequently, it is imperative to implement proactive strategies for smoking cessation to prevent the onset of osteoporosis within clinical settings. Furthermore, risk factors, including diabetes and the frequency of physical activity, should be closely monitored in individuals who both smoke and suffer from osteoporosis (26, 27).

The strength of this study lies in its substantial sample size and national representativeness, drawn from the NHANES database, which enhances the generalizability of our findings. Additionally, the use of a multivariate regression model to adjust for potential confounding variables has increased the reliability of the results.

This study has certain limitations. Although the NHANES provides reliable demographic and clinical variables, the absence of a systematically recorded fracture history during the analysis period may introduce residual confounding factors, as osteoporotic fractures are a key factor in mortality. Additionally, potential measurement errors in self-reported smoking status and the diagnosis of osteoporosis may affect risk estimation. Despite multivariable adjustments, unmeasured confounding factors such as genetic susceptibility and medication adherence may still exist.

To address these issues, future research should take the following steps: 1) include longitudinal fracture registration to clarify the temporal relationship between smoking, bone loss, fractures, and mortality; 2) verify exposure assessment through biochemical validation, such as confirming osteoporosis diagnosis with smoking cotinine levels and dual-energy X-ray absorptiometry (DXA); 3) explore mechanistic pathways using bone turnover markers (such as CTX and P1NP) and inflammatory biomarkers; and 4) conduct multicenter cohort studies or randomized trials to test comprehensive smoking cessation and anti-osteoporosis interventions.

The use of NHANES’ nationally representative data enhances the epidemiological generalizability of this study. This highlights the complex interplay between modifiable lifestyle factors and skeletal health outcomes. Our results provide a basis for redefining risk stratification paradigms and designing targeted interventions to overcome the smoking–osteoporosis–mortality axis. Further validation through biologically grounded longitudinal studies is essential to translate these associations into actionable clinical pathways.

## Data Availability

The datasets presented in this study can be found in online repositories. The names of the repository/repositories and accession number(s) can be found below: All data are available as publicly accessible datasets through NHANES. It is open and publicly accessible through the following link: https://wwwn.cdc.gov/nchs/nhanes/.

## Footnotes

1
https://www.cdc.gov/nchs/hannes/continuouschannels/. Please note that due to potential network issues or the link itself, the webpage may not be accessible. It is recommended to check the legitimacy of the web address and attempt to access it again.

2
http://www.R-project.org.
