# Supplementary material for: Associations between smoking and osteoporosis and all-cause mortality in participants from the United States: a cohort study
Source: Front Endocrinol (Lausanne). 2025 Mar 24;16:1533633. doi: 10.3389/fendo.2025.1533633 (PMC11973091; doi:10.3389/fendo.2025.1533633)
Supplement: Supplementary file 1 [file Table1.docx]

TABLE S 1 Univariate analysis

| Item | HR(95%CI) | P(Wald's test) |
| --- | --- | --- |
| Age (cont. var.) | 1.1 (1.09,1.1) | < 0.001 |
| Sex: female vs male | 0.71 (0.66,0.77) | < 0.001 |
| Race: ref.=Non-Hispanic White |  |  |
| Non-Hispanic Black | 0.76 (0.69,0.84) | < 0.001 |
| Other Race | 0.39 (0.35,0.44) | < 0.001 |
| BMI: ref.=18.5~24.99 kg/m2 |  |  |
| 25.00~29.9 kg/m^2^ | 0.9 (0.82,0.99) | 0.032 |
| ≥30.00 kg/m^2^ | 0.8 (0.72,0.88) | < 0.001 |
| Drink: yes vs no | 0.47 (0.43,0.5) | < 0.001 |
| DM: yes vs no | 2.72 (2.5,2.95) | < 0.001 |
| Hypertension: yes vs no | 3.67 (3.37,4) | < 0.001 |
| PFG (cont. var.) | 1.1 (1.08,1.11) | < 0.001 |
| ALB (cont. var.) | 0.87 (0.86,0.88) | < 0.001 |
| P (cont. var.) | 1.2 (0.96,1.49) | 0.102 |
| Ca (cont. var.) | 0.56 (0.36,0.86) | 0.009 |
| TG (cont. var.) | 0.99 (0.96,1.02) | 0.458 |
| TC (cont. var.) | 0.85 (0.82,0.88) | < 0.001 |
| HDL (cont. var.) | 1.07 (0.98,1.18) | 0.137 |
